# From Slow Shifts to Fast Flips: Unraveling problem-based learning group function dynamics

**DOI:** 10.1186/s12909-024-05542-8

**Published:** 2024-05-17

**Authors:** Matthew Mellon, Nicholas Dunn, Arden Azim, Teresa M. Chan, Matthew Sibbald

**Affiliations:** 1 Department of Medicine, Michael G. DeGroote School of Medicine, McMaster University, 1280 Main St. W, Hamilton, ON Canada; 2 McMaster Education Research, Innovation and Theory (MERIT) Program, Faculty of Health Sciences, 100 Main Street West, Hamilton, Canada; 3 School of Medicine, Toronto Metropolitan University, 350 Victoria St., Toronto, Canada

**Keywords:** Problem-based learning, Health professions education, Group function

## Abstract

**Purpose:**

Problem-Based Learning (PBL) relies on self-directed learning in small groups in the presence of a tutor. While the effectiveness of PBL is often attributed to the dynamics of group function, change in group function over time and factors influencing group function development are less understood. This study aims to explore the development of PBL group function over time to better understand the factors that give rise to high-functioning groups.

**Method:**

We examined time-function graphs of group function and conducted semi-structured focus group discussions in 2023 with medical students enrolled in a PBL curriculum. Students reflected on their experiences in four different PBL groups, creating time-function graphs to characterize development of group function over 8–12-week periods. We analyzed graphs and transcripts in a staged approach using qualitative description and direct content analysis, sensitized by two frameworks: Tuckman’s Stages of Group Development and the Dimensions of PBL Group Function.

**Results:**

Three archetypes of PBL group function development were identified: Slow Shifters, Fast Flippers, and Coasters. (1) Slow Shifters were characterized by a complex and extended pattern of growth consistent with Tuckman’s model, typically occurring amongst inexperienced groups, or groups faced with a novel task. (2) Fast Flippers were characterized by abrupt state changes in group function arising from internal or external disruptions. (3) Coasters were characterized by plateaus, where maintenance of group function was a frequently cited challenge. Abrupt changes and plateaus occurred more among mature groups and groups with significant PBL experience.

**Conclusions:**

PBL group function varies over time in 3 different patterns. Classic Tuckman’s stages are apparent among inexperienced groups, or groups facing novel tasks, whereas experienced groups often face abrupt change or plateaus. PBL educators and students should consider the need for novelty and disruption in more experienced groups to incite growth.

**Supplementary Information:**

The online version contains supplementary material available at 10.1186/s12909-024-05542-8.

## Introduction

Problem-based learning (PBL) is a pedagogical model widely used in health professions education (HPE), where small groups of students approach case-based learning in a social and collaborative manner [1–4]. The smallest functional unit of the PBL model is the group, as opposed to its individual members [5]. As such, high functioning groups are essential to the success of PBL [4–6]. While its structure varies, the principles underpinning the PBL process are consistent– groups develop learning objectives, formulate hypotheses, students engage in self-study, and report findings to their groups [7, 8]. An experienced facilitator or tutor is present to supervise and guide the group with minimal intervention [9, 10]. Groups work together over a period of weeks to months and engage in an iterative process of reflective discussion and group improvement throughout their course [11]. 

In the PBL model, the goals of knowledge acquisition and problem solving are equally as important as the interpersonal dimensions of the group. PBL initially emerged as a simulation of professional practice meant to mimic the social dynamics of health care teams [1, 2]. The social aspects and longitudinal interactions of PBL groups facilitate the acquisition of teamwork, cooperation, and communication skills, which enable groups to achieve a high level of functioning [4, 9, 12]. Student experiences and perceptions of learning are closely related to higher group function [4]. Thus, PBL groups must be able to reflect on and improve their functioning to enable their success. However, group function is heterogeneous and changes over time in an unpredictable manner [4]. In combination with the lack of a theoretical framework of PBL group function, it is difficult for groups to evaluate their function and to benchmark their progress in real time. If frameworks are available to better understand the unpredictable nature of group function development, PBL students and facilitators can work to optimize this during the PBL process.

Existing theories of group development are largely based on teams in organizational settings and are not specific to the unique context of HPE curricula [13]. However, they still provide a useful lens to analyze the factors the give rise to PBL group function development. Tuckman’s Stages of Group Development describe the development of group function as a slow stepwise process [14]. This theory characterizes group development as sequentially progressing through four stages: Forming, Storming, Norming, Performing [14]. The stages outline a process comprised of exploration, resistance to influence, then development of roles and social cohesion [14]. Only then do groups resolve outstanding issues and enter a culminative stage of high energy and functioning [14]. Tuckman’s model provides a generic framework of group behaviour grounded in the social nature of their interactions, but the extent to which it underpins PBL group development is not clear.

Tuckman’s theory can be applied to the PBL context using a well-defined model of PBL group function. Existing frameworks describing factors that influence PBL group function are summarized in Table 1 [15–18]. The Dimensions of PBL Group Function is one such framework that emerged from a scoping review of PBL group function literature and established four domains that describe the essential characteristics of highly functional PBL groups: the learning climate, facilitation and process, engagement and interactivity, and evaluation and group improvement [19]. While these domains offer a working definition of PBL group function, they do not characterize its dynamic nature and how it changes over a group’s course. It is also unclear how existing theories of group function development map onto PBL in the HPE context. As such, this study aimed to determine whether Tuckman’s model adequately characterizes the evolution of PBL groups and the factors that give rise to their evolution.

**Table 1:** Existing theories and frameworks of PBL group function

Author, Year	Study title	Core components
Schmidt and Moust (2000)	Factors Affecting Small-Group Tutorial Learning: A Review of Research	1) The role of problems 2) Cognitive processes 3) Intrinsic motivation 4) Influence of the tutor`
Hendry et al. (2006)	Group Problems in Problem-Based Learning	1) Quiet student 2) Absenteeism 3) Dominant student 4) Dismissal of psychosocial concepts 5) Disorganized process 6) Lack of commitment 7) Lack of tutor expertise 8) Personality clash 9) Superficial case engagement 10) Shortcutting process 11) Skipping concepts 12) Bullying
Azer and Azer (2015)	Group Interaction in Problem-Based Learning Tutorials: A Systematic Review	Tutor Factors 1) Perception of tutor role 2) Professional background 3) Group dynamic skills Student Factors 4) Previous training 5) Self-reflection 6) Peer feedback 7) Group setting Problem Factors 8) Problem characteristics
Fonteijn and Dolmans (2019)	Group Work and Group Dynamics in PBL	Resource Pool 1) Group size 2) Individual differences 3) Ability 4) Experience 5) Diversity Group Process 6) Learning task 7) Autonomy 8) Group climate 9) Team learning behaviours Learning Context 10) Academic discipline 11) Culture 12) Socialization and training 13) Tutor Structural Losses 14) Lack of elaboration 15) Common knowledge effects 16) Pressure for conformity 17) Unproductive brainstorm Interpersonal Losses 18) Social categorization 19) Poor adjustment to PBL 20) Unequal participation 21) Communication challenges 22) Time/routine problems 23) Absent tutor

## Methods

We conducted a mixed methods study combining pictographic drawings and focus group discussions using direct content analysis among medical students who have participated in a sequence of PBL groups.

### Study team

The study team was selected to promote diversity of perspectives, experiences, and educational backgrounds. The team included students, educators, and scholars of HPE and PBL, which enabled richer co-construction of the meaning derived from participant testimonies. MM is a medical student with experience in educational development and as a student in a PBL curriculum. ND is a medical student with experience in qualitative research and as a student in a PBL curriculum. AA is a General Internal Medicine fellow and Master of Health Professions Education candidate with experience in qualitative research. TC is a clinician scientist with experience in HPE as a leader and researcher. She is also the Dean of the School of Medicine and Vice President of Medical Affairs at Toronto Metropolitan University. MS is a clinician scientist with experience in PBL as a tutor, educator, and researcher. He is also the Associate Dean (Undergraduate) for the School of Medicine. MS had no direct involvement with participants to mitigate potential impact of his role on data collection.

### Context and participants

We conducted this study at the Michael G. DeGroote School of Medicine at McMaster University, where PBL is the foundation of the undergraduate medical curriculum. The pre-clerkship portion of the curriculum is subdivided into five Medical Foundations (MFs). Each MF is 8–12 weeks long and covers a specific set of content knowledge (ex. MF1 encompasses Respirology and Cardiology). Students are required to attend 2 PBL tutorials and 1–2 active learning large group lectures each week. Formal assessments are limited to 2–3 low-stakes concept application-based assessments during each MF. Each MF is one iteration of PBL, where students are placed in groups of 6–8 for the duration of the MF, then switched to groups with new peers for subsequent MFs.

We used a purposive recruitment strategy aimed at first- and second-year undergraduate medical students at McMaster University. We chose this cohort of prospective participants based on their unique experience participating in multiple successive iterations of PBL and the recency of their experiences. Eligible students were defined as (i) proficient in English, (ii) an undergraduate MD student enrolled at McMaster University, and (iii) having participated in at least four iterations of PBL in the MD program, each with a distinct group of students.

### Data collection

We collected data from participants at single meeting times, each consisting of two stages: an independent stage (time-function graph drawings and written annotations), directly followed by a group stage (semi-structured focus group discussions). Five sessions were conducted, each co-facilitated by MM and ND to maximize consistency and to reduce the potential influence of personal relationships between participants and a single co-facilitator. Each session was composed of 2–6 participants. At the start of each meeting, we asked participants to privately disclose to the co-facilitators whether they had previously shared a MF group with any other participants. As a result, the group was split in half and concurrent focus groups were conducted (one by each co-facilitator) for two sessions, with the shared group members split up. This was done to establish a safe environment for participants to openly share their views and experiences.

Written consent was obtained from each participant before the start of each session. The sessions were conducted virtually over Zoom® (San Jose, CA). The latest version of the focus group guide has been provided as Additional File 1. Since first-year students had not yet completed their fifth MF at the time the study was conducted, participants were asked to report only on their experiences over the first four MFs to maintain consistency. Prior to the focus groups, participants were asked to reflect on their group experiences using the Dimensions of Group Function as a sensitizing lens and a working definition of group function.

In the first stage of each session, participants drew line graphs depicting group function (y-axis) over time (x-axis) for each of four MFs. Participants also provided written annotations for up to five points on each graph to provide context for transformative moments in their groups’ trajectories. The participant worksheet has been provided as Additional File 2. Our choice of pictographic data draws from previous work on the use of “rich pictures” in qualitative HPE research. Visual methods of data collection are proposed to capture complexity that can be lost during the reductive process of simplifying participant testimonies [20]. Pictographic drawings should be coupled with participant testimonies, which we collected here via participants’ written and discussion-based inputs [21]. 

In the second stage of each session, participants engaged in semi-structured focus group discussions. We elected to conduct focus groups to provide participants the opportunity to contextualize their drawings and enable an exchange of ideas such that they could reflect on the similarities and differences between their group experiences. The co-facilitators asked participants to present one of their graphs and asked follow-up questions around similarities and differences between graphs and major turning points in group function. During each session, graphing worksheets were uploaded to a secure file directly by participants, de-identified, and compiled for analysis. Focus group discussions were audio recorded through Zoom® (San Jose, CA) and transcribed verbatim. Transcriptions were generated by a secure external service, Scribie® (San Francisco, CA). Transcripts were de-identified by the research team and focus group recordings were deleted. Our team reviewed transcripts in an iterative manner to evolve the focus group process and inform sampling.

### Data analysis

We reviewed the graph drawings, written annotations, and transcripts in a staged approach moving from qualitative description to direct content analysis [22]. Initial readings relied on a mixed approach of inductive and deductive coding to identify key themes using shared online documents, sensitized by two theoretical frameworks in a manner consistent with direct content analysis [22, 23]. The Stages of Group Development were used as a benchmark for analyzing time-function graphs, where graphs were assessed for congruence to this model [14]. The Dimensions of Group Function were used by participants as a definition of group function and by the research team as a list of potential influencing factors [19]. This framework was chosen since it is an amalgamation of several theories and captures a large scope of the existing literature. The four domains were used as axial codes, with the seventeen sub-items being introduced as additional codes only if they arose in the data. Both frameworks were used for deductive coding across all transcripts. Inductive coding was used for both time-function graphs and transcripts to identify unexpected and emergent themes. Additional codes for creating and maintaining group function, disruptions, plateaus, and diversity were added as these concepts emerged from the initial transcripts.

All members of our team discussed codes and linkages in regular meetings, with subsequent transfer of the group’s coding into qualitative analysis software, Dedoose® (Manhattan Beach, CA). This enabled an iterative transition to axial coding identifying more in-depth linkages between material. We continue this process until saturation was achieved, defined as empiric support for themes in line with our research objectives, consistent themes in spite of additional data, and meaningful impact for PBL educators and students [24]. 

### Approach to methodologic rigor

We adopted a systematic approach to rigor through data collection and analysis. Co-facilitators used a structured pre-briefing to establish expectations around confidentiality, and psychological safety for the focus group process. Transcripts were verified for accuracy and anonymity prior to analysis. The analytic team was constructed to balance perspectives including academics, leaders, and students with perspective into the PBL process. Analysis involved triangulating data across focus groups, examining multiple theoretical perspectives, and revisiting codes and themes in the analytic process. Coding was conducted in three distinct stages, allowing emergent perspectives to shift the approach to analysis. Reflexive practices were employed to acknowledge and neutralize harmful researcher bias while also leveraging individual subjectivity such that it gave rise to unique perspectives in data analysis [25]. Our team undertook reflexive journaling to identify emergent insights and wrote memos on shared focus group transcripts. The research team met on multiple occasions to discuss data impressions and engaged in structured team reflexive discussions. Two meetings occurred between focus groups to review preliminary data and evolve the focus group and data analysis processes. One meeting occurred after the final focus group to achieve consensus on final themes.

## Results

**Fig. 1:**
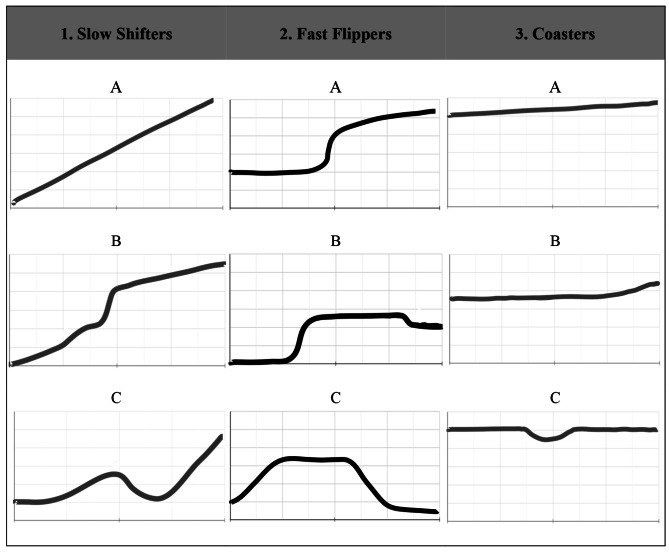
Three archetypes of group function development were identified. Archetypes are shown as time-function graphs drawn by first- and second-year medical student participants illustrating PBL group function (y-axis) over time (x-axis). Three representative examples of each archetype are provided. Written annotations have been removed. Data was collected at McMaster University in 2023

A total of 20 participants were recruited (14 first-year students and 6 second-year students, 10 male and 10 female) across five sessions ranging from 27 to 35 minutes. Based on students’ perceptions of their experiences, we identified three themes: (1) the experience-dependence of Tuckman’s model, (2) disruptions in group function give rise to state changes, and (3) a desire for comfort and status quo give rise to plateaus. From these themes, three archetypes of group function development emerged: Slow Shifters, Fast Flippers, and Coasters. Examples of time-function graphs drawings for each archetype are provided in Fig. 1.

Anchored by the theoretical frameworks, we identified four contextual factors on which the development of specific archetypes was most often dependent. Three emerged via deductive coding using Li’s Dimensions of Group Function and one emerged as a new insight via inductive coding. The contextual factors that give rise to each group function archetype are listed in Table 2.

**Table 2:** Contextual factors that give rise to group function archetypes

Dimensions of group function	Contextual factors	Slow shifters	Fast flippers​	Coasters​
​N/A	Level of PBL Experience	Associated with novel tasks (first-time groups); focus on creating function​	Associated with familiar tasks (subsequent groups); focus on maintaining function	Associated with familiar tasks and later in group course; focus on maintaining function
D1: Learning Climate​	Social Cohesion​	Optimal cohesion can boost morale and cooperation over the group’s course​	Critical group bonding moments can sharply increase function ​	Low cohesion leaves group members uncomfortable to make changes; High cohesion fosters complacency
D2: Facilitation and Process​	Tutor Influence​	Unique tutor perspectives can enrich group experiences	Critical events – autonomy struggle, learning vs. performing​	​N/A
D4: Evaluation and Group Improvement	Reflection and Feedback	Feedback is effective over the long-term and can repeatedly influence group function​	Feedback is effective in the short-term, typically at one defining event​	Feedback is mostly ineffective; the group is less willing to invest effort in development​

### Slow Shifters: The experience-dependence of Tuckman’s model

Groups whose trajectories followed Tuckman’s stages were uncommon and presented most often in MF1 groups (first-time PBL groups). These groups are labelled as Slow Shifters: they are defined by a complex process of steady growth that slowly tapers to a maximum level of functioning (Fig. 1: 1 A, 1B). While they may experience abrupt changes to their trajectories (Fig. 1: 1 C), their overall pattern of growth is typically gradual and continuous. This archetype was modulated by the novelty of the PBL context. Participants indicated that their earliest groups had a greater focus on the *creation* of group function; uncertainty around the PBL process fostered a greater propensity for exploration to improve group function through trial-and-error:

*I think the goals or the function change over time. So MF1, I think people are a lot more keen and eager, and they don’t really know what’s up. They don’t always know what’s the best learning, so they’re willing to try PBL a bit more. But I think there’s a lot more room or enthusiasm to change things up, versus as MFs progress, it’s a little bit more status quo and it’s like, ‘Oh, this worked in the past.* (Participant 6, Year 2)

The novelty of PBL enabled groups to be more responsive to other factors – tutor influence and social cohesion – such that they had a long-term benefit to group function development. This promoted the collective energy required to engage in the Tuckman-like process of ongoing reflection and improvement. One participant described a strong tutor who had the capacity to set a positive tone for group function:

*In MF1, I had JL, the cardiac surgeon for cardiology, and he’s so smart and so nice and kind, and he made the environment so good and he’d always ask us probing questions, and it was incredible.* (Participant 12, Year 1)

Participants also highlighted the impact of social cohesion. At an optimal level, personal connections gave rise to a progressively improving learning climate:

*When you start MF1, there is I think a little bit more confusion as to what PBL is and everything. But I feel like in my opinion, it just got better over time because you just get to know the other students well and become just closer with them as friends.* (Participant 6, Year 2)

The outcomes of reflection and feedback were reported as having highly inconsistent outcomes. However, participants reported feedback as being most effective when modulated by the factors listed above: the novelty of PBL, a strong tutor, and optimal social cohesion. As a result, groups with these characteristics could iteratively engage in reflection and implement feedback over their entire course, which enabled a Slow Shifter pattern:

*I’ve seen in groups where everybody wants to do feedback and the result is so much different than just having that idea of doing feedback and keeping it as like a check mark for the end of tutorial. [Be]cause I think in my MF1 group, it was something that we all really wanted to do and we all thought that every time we did it, it improved our functioning.* (Participant 16, Year 1)

### Fast Flippers: Disruption in group function gives rise to state changes

Many groups had flat trajectories, but experienced critical events, or *disruptions*, that gave rise to deviations from their natural course. Such deviations would not have otherwise occurred without an inciting factor. These groups are labelled as Fast Flippers: they are defined by a simple progression punctuated by disruptions that give rise to a sharp change in function. In contrast to the Slow Shifters, these groups are characterized by sudden state changes, with distinct before-and-after states (Fig. 1: 2 A, 2B, 2 C). Slow Shifters may experience disruptions (Fig. 1: 1B), but Fast Flippers usually do not experience significant growth or fluctuations outside of disruptions. Disruptions mostly have a positive impact on group function, but are sometimes negative (Fig. 1, 2 C). Here, participants indicated a greater focus on *maintaining* group function as groups became more experienced and familiar with PBL; they would establish a new status quo following the disruption.

Disruptions did not appear to be encouraged by aspects of the group environment, but rather arose spontaneously from events or deliberate actions by tutors or group members. Participants reported tutor interactions as giving rise to disruptions. One participant discussed an instance of a tutor setting expectations around assessments as a turning point for their group function:

*I think that after our first [assessment]… we had a talk with the entire group as well as the facilitator and what she was trying to get at is that these marks don’t mean anything…after hearing that, we kind of set our expectations…we really just started becoming more invested in our learning as opposed to getting superficial marks on the [assessments]. And I think the facilitator kind of pushed for that type of thinking.* (Participant 17, Year 2)

Participants also discussed social bonding moments as disruptions. In contrast to Slow Shifters, where social cohesion acts in a longitudinal manner, Fast Flippers experience critical moments of social cohesion:

*We went out for dinner as a group, and that was, I think, good, [be]cause it helped people get more comfortable with each other which then made tutorials run a little smoother.* (Participant 14, Year 1)

Participants discussed how critical moments of feedback and reflection gave rise to disruptions, but they typically did not benefit from feedback at other instances. This was due to their focus on group function *maintenance*, where there were few concerted efforts at actively improving group function outside of the disruption. This may be a result of infrequent feedback or feedback that does not get implemented. One participant discussed a group with a flat trajectory of group function outside of a dedicated moment of feedback:

*That was the only one where we had a dedicated tutorial skills tune up. I remember my group used that session to make it a full feedback session. I think after that we did make some changes, so we kind of exited a plateau.* (Participant 9, Year 1)

### Coasters: A desire for comfort and status quo gives rise to plateaus

The most frequently observed group function archetype was characterized by a persistent *plateau*, lacking substantive state changes or defining moments over their course. These groups are labelled as *Coasters*: they are defined by a constant level of group function with a clearly defined baseline. The baseline may be at a high (Fig. 1: 3A) or low level of functioning (Fig. 1: 3B). Groups may experience momentary deviations but differ from Slow Shifters and Fast Flippers in that they resist sustained inflections and return to their baseline (Fig. 1: 3 C).

The Coaster archetype is enabled by complacency and a resistance to change. These groups displayed experience-dependence in an opposite manner to Slow Shifters. Coasters, who usually had more PBL experience, reported complacency and satisfaction with their group’s function more often. They felt that group improvement was not needed or that their groups would not be responsive to efforts for improvement. Coasters resembled Fast Flippers in their emphasis on group function *maintenance*, but differed due to the absence of *disruptions*. Groups become comfortable and unwilling to invest the effort required for active group improvement:

*I thought, “Okay, we’re going to meet our objectives and we’re going to get those done and we want to get along and we’re going to address anything obvious.“…We were trying just to get good enough and then keep it there [be]cause we didn’t [want to] do extra work because we’re [all so] busy. So to me, the plateau was actually the objective.* (Participant 8, Year 2)

While the plateau defines the entire course of Coasters, this phenomenon was not unique to this archetype. Plateaus exemplified a time-dependence, where even the most actively changing Slow Shifters could experience relative stagnation late in their course (Fig. 1: 1B). The factors mediating plateaus in other archetypes are the same: complacency and resistance to change. Participants highlight end-of-group fatigue as a contributing factor:

*I feel like [that] was more reflective of the fact that we all sort of realized that there’s only a week or two left in the MF. We didn’t really put too much effort into really trying to optimize anything else. We were just fairly happy with where we were.* (Participant 10, Year 1)

Social cohesion and group feedback were found to be co-dependent in mediating resistance to state changes, typically in low-functioning plateaus. While Slow Shifters and Fast Flippers emerged from optimal levels of social cohesion, Coasters were characterized by inadequate social cohesion, which acted as a barrier to reflection and feedback:

*I have friends in groups that were way more dysfunctional, and I felt like that really stemmed from not getting along. That could not be resolved with feedback as well.* (Participant 6, Year 2)

Participants also cited too much social cohesion as a barrier to group function development through feedback:

*[Feedback] didn’t really work out the way we wanted it to [be]cause it was either like a lot of friends in a room who were never [going to] give negative feedback or a lot of people who were functioning negatively who didn’t actually [want to] be truthful about it… at this point, we had our mid MF feedback and it was all like, we’re all so great, we’re all best friends, this is so fun. And socially, it was fun, but learning-wise, it was not.* (Participant 13, Year 1)

## Discussion

Based on students’ perceptions of their group experiences, we identified three distinct archetypes of PBL group function development. Slow Shifters are groups who undergo a complex and sustained pattern of growth over their whole course; Fast Flippers are groups who have flat trajectories but experience sudden *disruptions* in their groups causing a discrete change in function, most often a positive change; Coasters are groups who do not experience meaningful growth or change and progress at a functional *plateau*. The plateau was a defining characteristic of the entire course of Coasters, but plateaus were observed in all archetypes towards the end of their course.

We found Tuckman’s Stages of Group Development to have crucial transferability in the PBL context, but its relevance was not universal [14]. The multi-step paradigm of this model was observed in Slow Shifters: groups with limited experience in a PBL context who possessed more energy for the exploratory process of group function *creation*. Experienced PBL groups (Fast Flippers and Coasters) did not display the classical model of developmental stages and instead displayed group function *maintenance*, except for abrupt but infrequent functional state changes. Hence, we propose that Tuckman’s model applies to inexperienced groups faced with a novel task but is inadequate to describe the development of experienced groups faced with familiar tasks. This form of group behaviour is explained by new models of group function characterized by *plateaus* and *disruptions*.

Our findings have several implications for educators, tutors, and students involved in PBL curricula, whose focus should be around group function improvement throughout the PBL process. The observation of Slow Shifters fits with our existing knowledge of group function development and does not warrant any modifications to the PBL model. It is the natural course of these groups to engage in continuous improvement without external influence, in a manner consistent with Tuckman’s model [13, 14]. The emergence of Fast Flippers and Coasters have a variety of implications. Experienced groups may not require a formal process or dedicated time for group function creation. Rather, they can establish a high level of function almost instantaneously after coming together. In contrast with Tuckman’s model, Forming, Storming, and Norming may not need to take place as these groups can move directly to Performing. While these groups demonstrate a predisposition to start out with a higher level of functioning, problems emerge as these groups progress since they are vulnerable to stagnation. This proves difficult to exit without a deliberate *disruption* to the group.

In the interest of consistent group improvement, the goal of experienced groups should be to identify the presence of plateaus and convert themselves from Coasters to Fast Flippers. We propose an optimal level of novelty, where educators should aim to ensure that sufficient novelty is present in experienced groups to enable disruption. Group composition should be shuffled frequently enough to avoid the adverse effects of high or low social cohesion. Since tutors can be catalysts for disruption, their effectiveness should be maximized through robust training and promoting student-to-tutor feedback. Educators can consider tutors with unique perspectives and professional backgrounds who may introduce novelty in group process. Evaluation and group improvement should be emphasized in the middle of these groups’ courses. This reflects their need for disruption later, as opposed to the less crucial process of early group forming. Feedback should focus on changes to group process, as opposed to reinforcing status quo.

Some limitations to this study and their implications on its transferability should be acknowledged. The retrospective nature of this study limits the recall capacity for participants and may fail to capture nuanced group interactions. As such, while we propose that experienced groups skip Tuckman’s stages of group development, it is also possible that they occur very rapidly and early in a group’s course such that the process is not memorable or remarkable enough to recall. An observational study would be required to verify these interactions, or lack thereof, in real-time. We also observed functionally stagnant groups who were unable to progress by engaging in feedback, but the reasons for why feedback was not effective were unclear. Future investigations should be conducted to better characterize the contextual factors that give rise to effective feedback. This study was conducted in the context of a PBL curriculum where students participate in multiple iterations of PBL with a relatively consistent task across groups. Therefore, the results may not be fully transferable to contexts where experienced individuals approach vastly different tasks between groups. We also did not investigate the influence of participants’ previous group experiences on subsequent groups, nor whether individual differences in perception were present within the same groups. Additional questioning would be required to understand how such individual experiences impact the perception of group function.

## Conclusion

The effectiveness of PBL relies on strong group function, but the development of PBL group function in complex and heterogeneous. PBL group function develops in three unique patterns, moderated by experience level and novelty of the context. Inexperienced groups new to the PBL context undergo consistently increasing patterns of group function development consistent with the classical stages of group development. Experienced groups demonstrate complacency and evolve along one of two possible trajectories: a sustained plateau over their entire course, or a plateau punctuated by instantaneous state changes in their function. In order to enable consistent growth and development in experienced groups, educators should consider the need to introduce novelty to the PBL context by means of challenging social factors, tutor variability, and intentional reflection and feedback.

### Electronic supplementary material

Below is the link to the electronic supplementary material.

Supplementary Material 1

Supplementary Material 2

## Data Availability

The datasets used and analyzed during this study are available from the corresponding author on reasonable request.

## Abbreviations

PBL: Problem-based learning
HPE: Health professions education
MF: Medical foundation
