# Supplementary material for: From Slow Shifts to Fast Flips: Unraveling problem-based learning group function dynamics
Source: BMC Med Educ. 2024 May 17;24:552. doi: 10.1186/s12909-024-05542-8 (PMC11102218; doi:10.1186/s12909-024-05542-8)
Supplement: Supplementary file 1 — Supplementary Material 1 [file 12909_2024_5542_MOESM1_ESM.pdf]

How PBL Groups Evolve – Participant Worksheet

- Take a moment to reflect on your group's function and its trajectory over the course of the MF.
- Plot a smooth, curved line graph using the axes below, where the X axis represents time, and the Y axis represents group function. Use the X-axis labels as approximations to orient yourself.
- Indicate the major group function "turning points" by drawing points along the line. Label up to 5 turning points and label them A-E.
- Using the chart below, describe each turning point using a single word, a few words, and/or a short sentence.

MF:____________

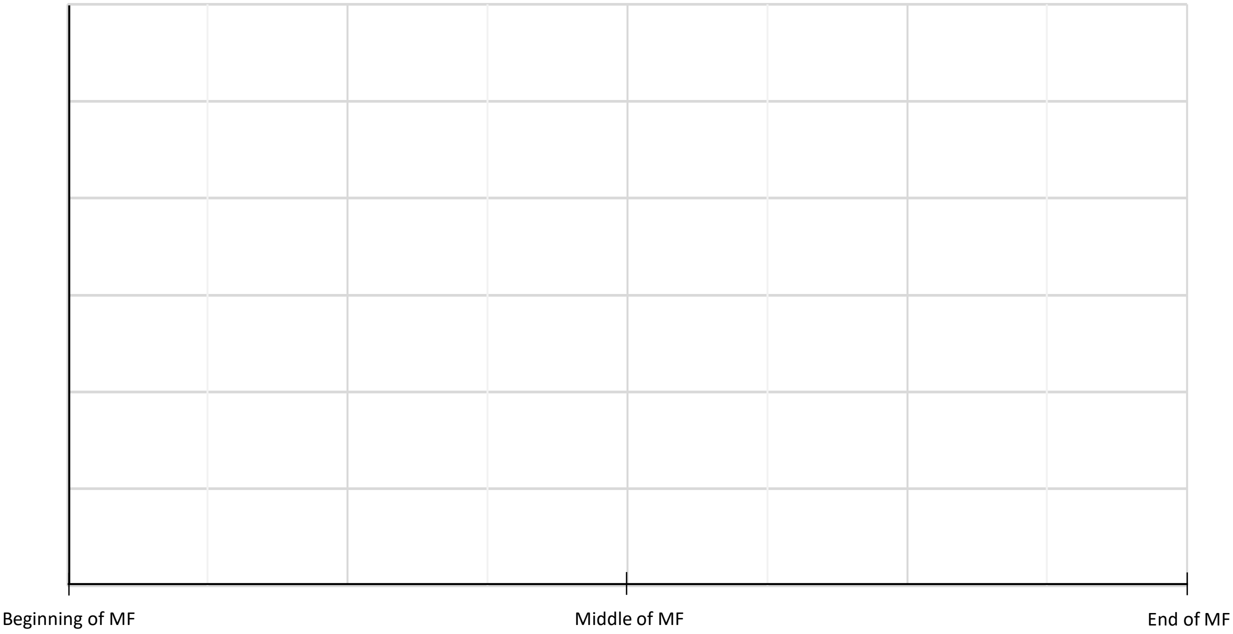

**A**	
**B**	
**C**	
**D**	
**E**	
