# Supplementary material for: From Slow Shifts to Fast Flips: Unraveling problem-based learning group function dynamics
Source: BMC Med Educ. 2024 May 17;24:552. doi: 10.1186/s12909-024-05542-8 (PMC11102218; doi:10.1186/s12909-024-05542-8)
Supplement: Supplementary file 2 — Supplementary Material 2 [file 12909_2024_5542_MOESM2_ESM.pdf]

Focus Group Guide for Participants

PLEASE NOTE: This is a focus guide, not a script. The exact wording and order of questions may change throughout the project and will be guided by the flow of each focus group. In general, the subjects of discussion will remain around participant's thought process during the graphing exercise, the nature of their groups' evolution over time, their group successes and challenges, and their perceived impact on PBL outcomes.

Introduction (5 mins)

DO NOT START W/O CONSENT FORMS

Our project seeks to understand **how group function changes** over the course of your PBL experience. With the recent development of a **tool to assess group function**, we now have a coherent set of descriptive language to discuss what group function means, and what it looks like in practice. However, this only tells us about **what constitutes group function at a point in time**. We still do not know how group function **plays out in real time**. For example, how does it evolve over a group's lifespan? Are there patterns between groups of this evolution? What are the successes and challenges that change the course of a group's function?

This is the reason for the initiation of this study: we are hoping to collect data on group function from all your previous PBL experiences and to analyze this data to understand the variation across groups. Our end **goal is to develop common trajectories of group function** that exist in PBL groups and to characterize common successes and challenges that give rise to strong or poor group function. We hope that this will improve the PBL experience by providing a model of strong group function for students and tutors to apply to their own groups.

Ask participants to send a private message to the facilitator(s) if they have shared an MF group with any other focus group participant. In this case, split up the group between the two facilitators to make sure that people from the same group don't have to participate together.

Stage 1: Independent Reporting (15 mins)

[Participants will be provided with line graph templates and an example of the graph format will be shown]

The idea of this exercise is to **draw four line graphs**, each of which depicts your group's function over time. The x-axis should depict the timespan of your group – the actual points of the graph do not need to follow a set scale but should represent standard segments of time (for example, dividing the timespan into two halves). The y-axis should represent group function – use the language from the assessment tool to reflect on how strongly your group functioned at each point in time, then set the graph's amplitude accordingly.

As you draw your graphs, consider the **inflection points of the graphs** – what happened within your group to change the course of your group's function trajectory? **Annotate your graphs** with a short

description of such instances. There is no recommended number of annotations, but space is provided for up to five.

Stage 2: Focus Group Discussion (40 mins)

REMIND TO SUBMIT WORKSHEET TO DROPBOX

Let them know that we may give directing questions and/or call on certain people to contribute

START RECORDING

1) Would any participants like to walk us through one of their graphs?
2) Did any other participants' graphs have a similar trajectory? Did any participants have drastically different graphs?
3) What are some commonalities between how each of your four groups change over time? What are some differences?
4) Did your subsequent groups influence your perception of your previous groups? If so, how?
5) What happened during some of the major turning points reported on your graphs? Were there specific *disruptors* (positive or negative) that altered the trajectory of your group function? What situational context allowed disruptors to occur? What happened after a disruption? How did the group handle it?
6) Did any other participants also experience similar disruptors?
7) How do you think diversity of thought/of resources/of contributions impacts group function?

- **If a problematic group member is brought up** → What is the connection between a problematic group member or tutor and poor group function? If you subbed out the problematic individual for someone else, how would that change the group's function? Would it change at all?
- **If group function is attributed to one factor only** → Was this one factor responsible for strong or poor group function, or are other factors involved as well?
- **If they were unsatisfied with their group function** → What did you do when you began to be unsatisfied with your group's function?
- **If group feedback comes up in discussion** → Does the timing of feedback matter for it to impact group function? What made people comfortable offering transformative feedback?
- **If a "plateau" is discussed on the graph** → What do you think contributed to this plateau? Did your group change the way it did feedback towards the end of the MF? Do you think it was possible for your group to exit the plateau (positively or negatively)?

Conclusion and Questions (2 mins)

Those are all the questions I have for you all right now. Did we miss anything? What questions do you have for me?
